# Subject Identity Confounds qEEG Emotion Recognition on DEAP and DREAMER

**DOI:** 10.3390/s26175327

**Published:** 2026-08-22

**Authors:** Ema Pandilova, Aleksandar Stojmenski, Ivan Chorbev, Marko Petrov, Ivan Kitanovski, Dimitar Trajanov

**Affiliations:** Faculty of Computer Science & Engineering, Ss. Cyril and Methodius University, 1000 Skopje, North Macedonia; ivan.chorbev@finki.ukim.mk (I.C.); marko.petrov@finki.ukim.mk (M.P.); ivan.kitanovski@finki.ukim.mk (I.K.); dimitar.trajanov@finki.ukim.mk (D.T.)

**Keywords:** qEEG, emotion recognition, valence, arousal, participant-independent evaluation, individual differences, personalized affective computing, DEAP, DREAMER

## Abstract

Quantitative EEG features such as frontal alpha asymmetry, spectral ratios and signal-complexity measures are often presented as interpretable biomarkers of emotion. Such claims require the markers to generalize across individuals, yet common evaluation protocols allow overlapping epochs and recordings from the same participants to appear in both training and test sets. We re-evaluated qEEG-based valence and arousal recognition on DEAP and DREAMER under trial-grouped, participant-independent, within-participant and cross-dataset protocols. Epoch-pooled evaluation on DEAP gave ROC-AUC values of 0.689 for valence and 0.711 for arousal, whereas participant-independent evaluation of the same features and model returned 0.493 and 0.447. Grouping epochs by trial accounted for about 0.06 of that difference and separating participants for a further 0.13 to 0.17. The same features identified participants with accuracy of 0.998 on DEAP and 0.891 on DREAMER, and a predictor that used no EEG, assigning each trial its participant’s training-set positive rate, accounted for 42 to 84 percent of the above-chance discrimination of the epoch-pooled model. Emotion-related effects were reproducible within participants on DEAP but close to zero on DREAMER, and their direction reversed for about 40 percent of features across participants. In a matched participant-level comparison using a single fixed estimator in both arms, training on a participant’s own data improved DEAP valence by 0.092 AUC (95% CI 0.029 to 0.157, Holm-adjusted p=0.042) and gave no reliable benefit for DEAP arousal or for either DREAMER target. Across the channels shared by the two datasets, per-feature arousal effect sizes correlated moderately, although no individual feature reached false-discovery-rate significance in both datasets. Pooled qEEG emotion-recognition scores can therefore reflect participant-specific recording structure rather than transferable affective information. Population-level claims require participant-independent evaluation, while personalization should be considered only where stable within-person effects are demonstrated.

## 1. Introduction

Emotions are a fundamental component of human psychophysiology, shaping behavior, modulating cognition, and guiding social interaction [1]. Because they are expressed and experienced across cultural and linguistic boundaries [2], the objective recognition of affective states has broad scientific and clinical value, from mental-health monitoring to adaptive human–computer interfaces.

Electroencephalography (EEG) allows brain activity to be studied objectively and with high temporal resolution [3]. It records the electrical activity of the cerebral cortex and resolves the classical rhythms: delta (δ, 1–4 Hz), theta (θ, 4–8 Hz), alpha (α, 8–13 Hz), beta (β, 13–30 Hz), and gamma (γ, 30–40 Hz). Unlike behavioral measures based on facial expression, speech, or posture, which can be masked or shaped by social context [4], EEG captures cortical activity that is not under full voluntary control and is therefore attractive as a more direct index of emotional state.

Quantitative EEG (qEEG) reduces the raw signal to numerical descriptors such as absolute and relative band power, band ratios (for example, θ/β and θ/α), frontal alpha asymmetry, connectivity indices, and complexity measures including entropy and fractal dimension [5]. These quantities are used clinically to assess cognitive and psychological states such as attention deficits, anxiety, and depression [6], and they are increasingly proposed as interpretable biomarkers of emotion. A biomarker, however, is useful only if it is reproducible in the population to which it is applied. Whether qEEG affective markers genuinely generalize across individuals and recording contexts is seldom tested.

Most studies report performance under evaluation protocols that pool many overlapping epochs from the same participants into a single cross-validation and that treat those epochs as independent samples in significance testing. Both choices can make a weak, participant-specific signal appear strong and general; such evaluation leakage is a recognized cause of inflated performance across machine learning fields [7,8].

This paper re-examines interpretable qEEG features for valence and arousal under a deliberately conservative, leakage-free protocol on two independent datasets, DEAP [9] and DREAMER [10]. Recent work has begun to document inflated accuracy in EEG-based emotion recognition [11]; here we go further and ask where the inflation comes from and what, if anything, survives once it is removed.

Using identical features and models, we decompose the optimism on DEAP under the pooled out-of-fold metric. Grouping epochs by trial removes near-duplicate windows and lowers the receiver operating characteristic area under the curve (ROC-AUC) by about 0.06; additionally separating participants removes identity overlap and lowers it by a further 0.13 for valence and 0.17 for arousal. Treating overlapping epochs as independent samples also overstates statistical significance, by more than ten orders of magnitude in the most extreme cases, relative to trial- and participant-level analysis. We then show that the apparent performance reflects participant identity more than emotion. A predictor that uses no EEG, relying only on a participant’s label tendency, reaches an ROC-AUC of 0.60 to 0.68. That accounts for 42 to 84 percent of the epoch-pooled model’s above-chance discrimination. The same features identify the individual with very high accuracy, reaching 0.998 on DEAP and 0.891 on DREAMER, while participant-independent emotion decoding is near chance. Participant identity also explains three to four orders of magnitude more feature variance than emotional state on DEAP and two to three on DREAMER.

Where a genuine affective signal exists, it is idiosyncratic. Within-participant effects are reproducible across split halves on DEAP, yet their direction reverses for about 40 percent of features across participants, and no feature that survives correction in DEAP survives it in DREAMER. Because the signal is individual, a model trained on a participant’s own data outperforms one trained on all other participants for DEAP valence, whereas the same matched comparison is null for DEAP arousal and for both DREAMER targets, which makes personalization a conditional rather than a universal remedy. To support reuse and scrutiny, we release a fully reproducible pipeline reporting effect sizes and confidence intervals, feature-selection stability, importance triangulation, feature-family and channel ablations, and matched within-participant, participant-independent, calibrated and cross-dataset evaluations.

This study uses an interpretable qEEG feature set and a compact fronto-parietal montage, with evaluation protocols designed to distinguish participant-specific structure from transferable affective information.

The remainder of this paper is organized as follows: Section 2 reviews related work and positions the study. Section 3 describes the datasets, features, protocols, and statistical methods. Section 4 reports the results, Section 5 discusses their implications and limitations, and Section 6 concludes.

## 2. Related Work

We review prior work along two lines: feature-based and deep learning emotion recognition on DEAP and DREAMER and the broader literature on generalization across participants, interpretability, and feature stability that motivates the present evaluation.

### 2.1. Feature-Based and Deep Learning Approaches

Early emotion-recognition studies on DEAP used hand-crafted spectral descriptors with simple classifiers. The dataset paper in [9] reported single-trial accuracy near 0.58 for valence and 0.62 for arousal using power-spectral-density features and a Gaussian naive Bayes classifier and established the binary low/high task adopted by most later work. Subsequent studies examined which features and selection strategies are most discriminative. Atkinson and Campos [12] combined minimum-redundancy–maximum-relevance selection with kernel classifiers and reached roughly 0.73 for both dimensions, while comparative analyses of feature families [13] and of stable spectral patterns across sessions [14] mapped the relative value of spectral, statistical, and connectivity descriptors, and the survey by Alarcao and Fonseca [15] consolidated these efforts. More recent hand-crafted pipelines report higher figures, for example, differential entropy and fractal dimension with tuned gradient boosting near 0.89 valence and 0.88 arousal [16], with related evaluations and feature-selection comparisons reporting similar gains [17,18,19].

A parallel line of work learns representations directly from raw or minimally processed EEG. Recurrent models were applied first, with Alhagry et al. [20] reporting around 0.85 for both dimensions using a long short-term memory network. Hybrid convolutional–recurrent and three-dimensional convolutional designs followed [21,22], and more recent architectures report still higher accuracy with participants shared between training and test data, including the transformer-capsule network by Wei et al. [23] near 0.99, alongside other convolutional and attention-based models [24,25,26,27,28]. Large self-supervised EEG foundation models such as LaBraM [29] and BIOT [30] extend this trend. These models report high absolute accuracy but offer limited insight into which cortical signals drive a prediction. For the present work it is relevant that most of these figures are obtained with participants shared between training and test data and often under epoch-pooled protocols, a point we quantify in Section 4.

### 2.2. Generalization Across Participants, Interpretability and Stability

EEG is strongly non-stationary across individuals, so a model trained on one set of participants typically degrades on unseen participants. This has motivated substantial research on transfer learning and domain adaptation, surveyed by Wu et al. [31], with foundational multi-source approaches such as style-transfer mapping by Li et al. [32]. Euclidean Alignment [33], which recenters each participant’s trial covariances to a common reference without requiring target labels, is a simple and widely used device that we adopt as a baseline. Work targeting emotion specifically includes the contrastive cross-dataset model by Liao et al. [34] and the multi-dataset pre-training by Zhang et al. [35], which aligns feature statistics across several corpora to support few-shot and zero-shot transfer. Foundation models reviewed in recent surveys [36,37] and inference-time adaptation methods [38] pursue similar goals.

A related concern is interpretability and the stability of the markers that a pipeline selects. Post hoc explanation methods [39], surveys of explainable methods for EEG [40], and frameworks for interpretable affective computing [41] reflect a shift toward inspectable models, while methodological work on selection stability [42,43] and feature-selection comparisons on DEAP [18] show that the selected subset can vary considerably with the method, so predictive accuracy alone does not guarantee reproducible markers.

A third strand addresses generalization from limited or heterogeneous observations, the regime in which affective EEG operates. The few-shot multimodal sentiment framework by Zhu et al. [44], which combines integrated prompt learning with vision–language models, is representative. Although it addresses social-media sentiment rather than EEG, it illustrates that transferable affective representations are usually obtained by injecting structure from large pre-trained models or auxiliary modalities, rather than by expecting a compact hand-crafted feature set to generalize on its own. A complementary observation comes from peripheral physiology. Long et al. [45] report participant-invariant emotional information from cardiac signals acquired using a photonic sensing system. Although that study concerns cardiac rather than EEG signals, it provides complementary evidence that participant-invariant affective information may be recoverable from other physiological modalities and a useful contrast with the strong participant-specific structure of the qEEG descriptors examined here.

Across these strands, prior work largely optimizes accuracy and rarely tests whether the qEEG indicators it relies on generalize across participants and datasets, since reported successes are usually obtained with participants shared between training and test data or under epoch-pooled protocols. The present study addresses this gap by asking, under a leakage-free protocol on two datasets, whether interpretable qEEG markers are reproducible across people and contexts. Table 1 positions representative DEAP studies against this work and makes the evaluation protocol explicit.

Reported participant-independent results on DEAP are markedly lower than the figures above, which share participants between training and test data, typically 0.67 to 0.68 for STILN [47], 0.65 to 0.70 for the cascaded self-supervised model by Wang et al. [46], and near chance in the unified re-benchmark by Kukhilava et al. [11], at 0.51 to 0.53 for valence and 0.53 to 0.57 for arousal, where every model evaluated falls below the trivial majority-class baseline. The present results fall within this realistic range, and we perform comparisons only within matched protocols.

## 3. Materials and Methods

This section specifies the analysis pipeline so that the reported results can be reproduced. We describe the two datasets and their preprocessing, the spectral and nonlinear qEEG features with their hyperparameters, the evaluation protocols, and the classifiers and statistical analyses. Unless stated otherwise, the same feature definitions and protocols are applied identically to DEAP and DREAMER.

### 3.1. Datasets and Preprocessing

**Figure 1:**
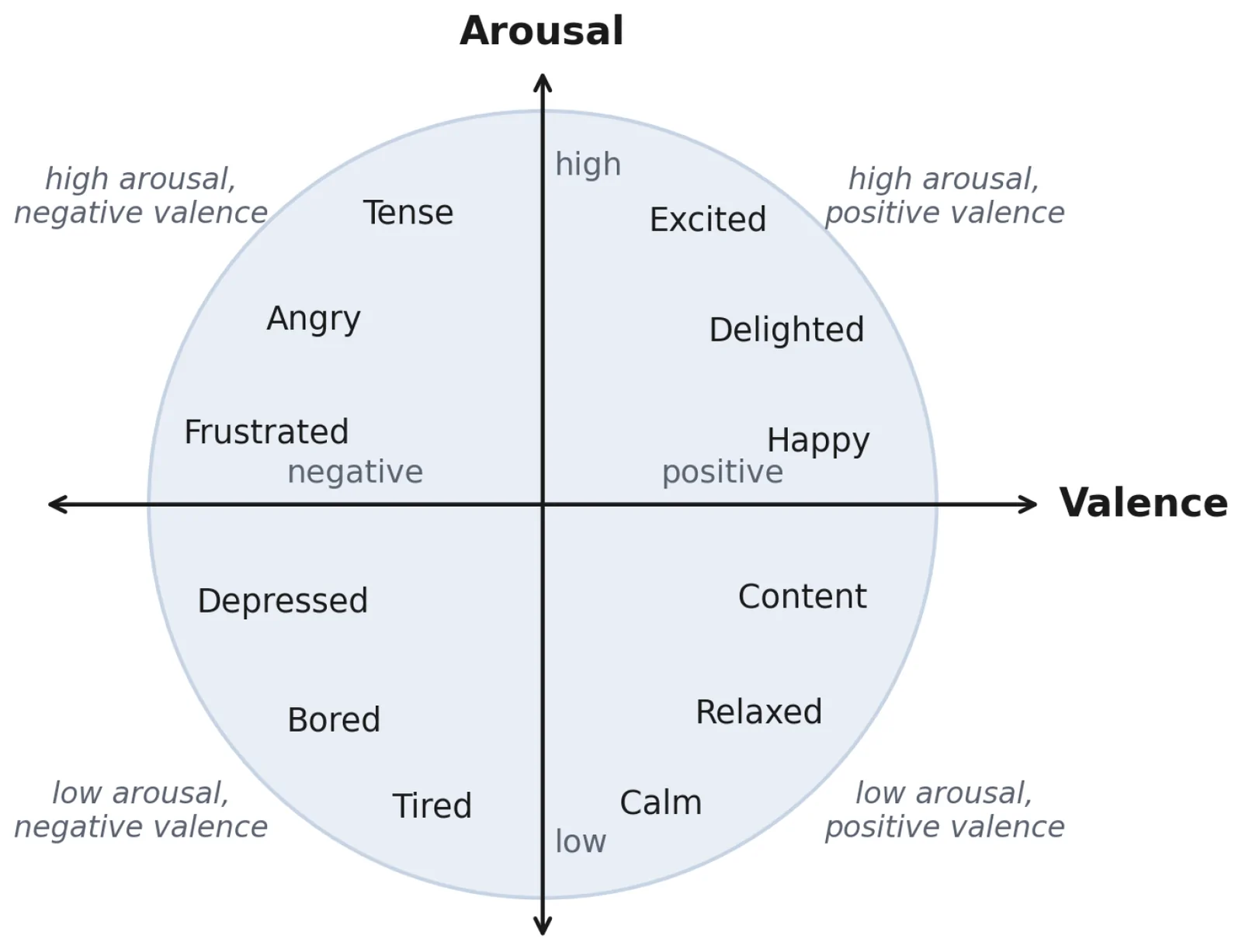
The circumplex model of affect, with valence on the horizontal axis and arousal on the vertical axis.

DEAP [9] is a public multimodal database of affective responses. It contains recordings from 32 participants (16 males and 16 females; ages of 19 to 37, with a mean of about 26.9 years), each of whom watched 40 one-minute music videos. EEG was recorded from 32 channels in the international 10–20 system, together with 8 peripheral channels (electrooculogram, electromyogram, galvanic skin response, respiration, temperature, and blood-volume pulse). After each clip, participants rated valence, arousal, dominance, and liking on nine-point Self-Assessment Manikin scales [48]. We focus on valence and arousal, following the circumplex model of affect [49] (Figure 1), and use the preprocessed release (EOG correction, 128 Hz, bandpass filtering). Valence and arousal labels were derived from the per-trial self-assessment ratings provided with the dataset.

DREAMER [10] is an independent affective database recorded with low-cost wearable hardware. Twenty-three participants watched 18 film clips while 14-channel EEG (Emotiv EPOC, 128 Hz) and ECG were recorded, and they rated valence, arousal, and dominance on five-point scales. DREAMER differs from DEAP in population, stimuli, montage, and acquisition hardware, which makes the pair a demanding test of cross-dataset generalization. The four EEG channels common to both datasets (AF3, AF4, F3, and F4) are used for all cross-dataset analyses. Table 2 summarizes both datasets.

The preprocessed DEAP signals (EOG-corrected and downsampled to 128 Hz) were used. Each 63 s recording contains a 3 s pre-trial baseline followed by the 60 s stimulus; the baseline was discarded, and all analyses use the 60 s stimulus interval. For DREAMER we use the last 60 s of each stimulus. A zero-phase fourth-order Butterworth bandpass (0.5 to 40 Hz) was applied, and the signal was segmented into 4 s epochs with 2 s (50%) overlap, giving 29 epochs per trial and 37,120 epochs for DEAP. No artifact rejection beyond the EOG correction already present in the preprocessed release was applied, so we make no further artifact-removal claim. The 50% overlap is stated explicitly because, as Section 4 shows, overlapping epochs make epoch-level pooling a strong source of optimistic bias.

### 3.2. Feature Extraction

**Figure 2:**
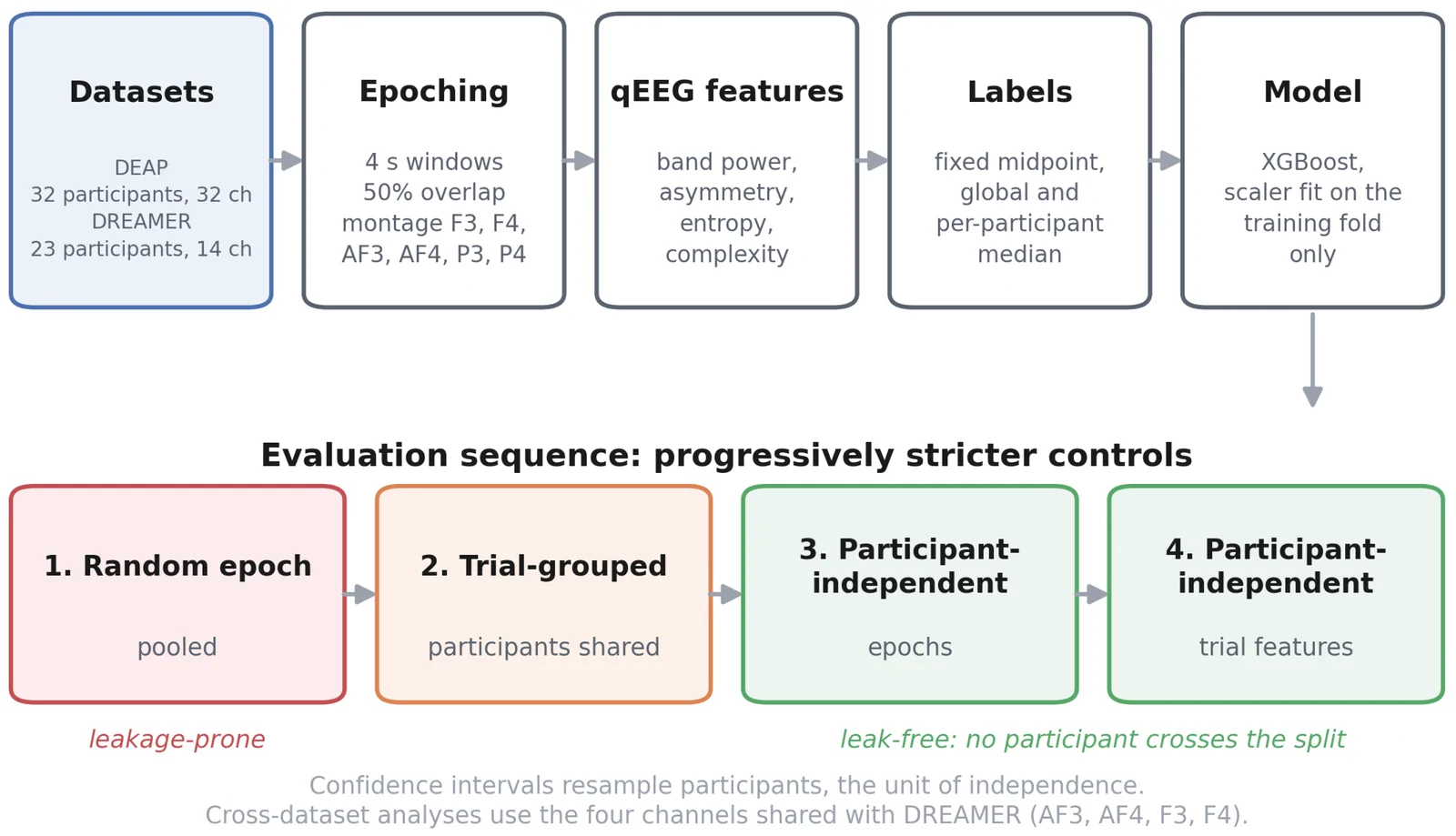
Overview of the EEG processing and analysis pipeline.

For every epoch and channel we computed a fully specified set of spectral and nonlinear qEEG descriptors, with all hyperparameters listed in Table A1. Spectral power was estimated with Welch’s method (Hann window, segment length equal to the epoch, 50% overlap) over five bands (δ 1–4, θ 4–8, α 8–13, β 13–30 and γ 30–40 Hz), as absolute and relative (band divided by total 1–40 Hz) power, together with the θ/α and θ/β ratios. Nonlinear descriptors were permutation entropy (embedding orders 3, 5 and 7; delay 1; normalized), Lempel–Ziv complexity (median-threshold binarization, normalized) and the three Hjorth parameters. Sample entropy and Higuchi fractal dimension were part of the extraction specification but returned a single constant value for every epoch of both datasets, so they carry no information and are excluded from all analyses reported here; the corresponding columns are reported as excluded in Table A1 rather than treated as descriptors. Frontal and parietal alpha log-asymmetry, $\ln P_{\alpha }^{\mathrm{right}}-\ln P_{\alpha }^{\mathrm{left}}$, was computed for the (F4, F3), (AF4, AF3) and (P4, P3) pairs. For the 32-channel configuration we additionally computed inter-channel phase-locking value and magnitude-squared coherence per band (Hilbert phase, second-order Butterworth band filters) for a fixed set of fronto-parietal and inter-hemispheric pairs, together with all-pair band log-asymmetries. After excluding the degenerate columns, the compact montage (F3, F4, AF3, AF4, P3, P4) yielded 117 informative features, the four channels shared with DREAMER yielded 78, and the full 32-channel set yielded 686. From DEAP’s peripheral channels we additionally extracted interpretable autonomic features per trial (heart rate and heart-rate variability from blood-volume pulse, electrodermal level and skin-conductance responses from GSR, respiration rate and variability, zygomaticus and trapezius EMG power, and skin-temperature statistics) for the multimodal analysis. Figure 2 summarizes the pipeline.

### 3.3. Label Binarization and Evaluation Protocols

DEAP valence and arousal lie on a nine-point scale and DREAMER on a five-point scale, and we treat binarization as an explicit design choice. Four schemes are compared: the fixed midpoint, the global median, the per-participant median, and a margin scheme that retains only trials whose rating is at least two points from the midpoint. Thresholds follow each dataset’s own scale, so the fixed midpoint is 5 for DEAP and 3 for DREAMER. The per-participant median splits a participant’s own ratings and therefore defines a within-person relative-state target rather than an absolute one, and because it uses that participant’s complete rating distribution, it presupposes label information that is unavailable before it has been recorded.

We therefore recommend the fixed midpoint for claims about absolute or population-level affect and reserve the per-participant median for questions that are explicitly about within-person relative state. The choice rests on construct validity rather than performance: the participant-independent AUC varies by less than 0.04 across the four schemes (Table 3). The fixed midpoint is accordingly used for protocol decomposition, the identity analyses and the matched personalization comparison, and the per-participant median where the question is within-person by construction. The sweep was run on DEAP with XGBoost under GroupKFold, so that comparison is specific to those choices.

Table 4 summarizes the evaluation protocols under which the main results are reported and assigns each to one of four classes. Leakage-prone protocols place epochs of the same trial, or recordings of a participant who also appears in training, on both sides of the boundary, so an estimator can recognize the test material rather than the affective state; they are reported only to quantify the bias they introduce. Inductive protocols estimate every fitted transformation and model parameter from training data alone. Transductive protocols let no label cross the boundary but estimate the standardizing statistics from the held-out participant’s own unlabeled recordings; per-participant standardization therefore constitutes a transductive protocol. Adaptive, or calibrated, protocols additionally use a small number of labeled trials from the target participant, as in the *k*-shot setting. Leakage is defined relative to a participant-independent claim, so the within-participant and participant-adaptive protocols, which use the target participant’s own data to support within-person or calibrated claims, are not leakage-prone in that sense.

**Table 4:** Evaluation protocols used in this work. Protocols are classified according to participant overlap, trial overlap, normalization strategy, and use of target-participant information.

Protocol	Participants Shared?	Epochs of a Trial Split?	Normalization	Class	Reported in
Random epoch pooling	yes	yes	train-fold	leakage-prone	Table 5, Table 6, Table 7 and Table 8
Trial-grouped, participants shared	yes	no	train-fold	leakage-prone (identity)	Table 5, Table 6, Table 7 and Table 8
Participant-independent, train-fold standardization	no	no	train-fold	inductive	Table 5, Table 6 and Table 7
Participant-independent, per-participant standardization	no	no	per participant	transductive	Table 3 and Table 9, Appendix A
Within participants (personalized)	one participant	no	train-fold (within participant)	inductive within participant	Table 12
Participant-adaptive (*k*-shot)	partly: *k* labeled calibration trials only	no	per participant	adaptive (calibrated)	Table A7
Cross-dataset transfer	no (other cohort)	no	per participant	transductive	Table A6

In the within-participant protocol, each participant is analyzed separately with cross-validation over that participant’s own trials. In the participant-independent protocol we use leave-one-participant-out and ten-fold GroupKFold with participants as groups, on trial-level features, which is the unit at which labels are defined; where epoch-level features are used, the epochs of a trial are kept together, and epoch probabilities are averaged to the trial before scoring. In the participant-adaptive protocol, a participant-independent model is augmented with *k* labeled trials from the held-out participant, for *k* from 0 to 20. The epoch-pooled design used in earlier work is reported only to quantify the bias that it introduces. We also evaluate cross-dataset transfer on the four channels shared by DEAP and DREAMER, training on one dataset and testing on the other. As a stronger domain-adaptation baseline we applied Euclidean Alignment [33] to per-trial band-limited covariance matrices, followed by Riemannian tangent-space projection and a linear classifier.

### 3.4. Classifiers and Statistical Analysis

We compared a majority-class baseline, logistic regression, linear and radial-basis-function support vector machines, random forest, and XGBoost, all with fixed hyperparameters. Class imbalance was handled using balanced class weights. Decision thresholds were selected with Youden’s *J* on an inner participant-aware validation split. As an additional check on the achievable performance ceiling, we also evaluated a nested participant-aware hyperparameter search with mutual-information top-*k* feature selection and a soft-voting ensemble. ROC-AUC was the primary metric, with precision–recall area under the curve (PR-AUC), balanced accuracy, per-class recall, and F1 also reported. Main performance estimates are accompanied by 95% confidence intervals obtained by bootstrapping over participants.

Model-family results are summarized as ranges because pairwise comparisons between families were not multiplicity-corrected and their ranking was not consistent across targets. When a single configuration is reported, it represents the highest value within the stated set and should therefore be regarded as an optimistic summary.

The comparison between within-participant and participant-independent modeling used a fixed matched design. Both arms used logistic regression with C=1, balanced class weights, identical features, the same trial-level representation and target definition, and no threshold tuning. The training protocol was therefore the only difference between the two arms. One ROC-AUC was calculated for each participant under each protocol, and the within-participant difference was summarized using a participant-bootstrap confidence interval and a Wilcoxon signed-rank test. No model-family selection was involved in this comparison.

Because epochs from the same participant and trial are not independent, statistical analyses were considered at the epoch, trial, and participant levels. Epoch-level results are reported only to illustrate the inflation that arises when non-independent observations are treated as independent. Trial-level analyses use the 1280 DEAP trials. For participant-level paired analyses, per-class feature means are first calculated within each participant and then compared across participants using the Wilcoxon signed-rank test. Effect sizes are reported as $\eta^{2}$ for ANOVA, $\epsilon^{2}=\left(H-k+1\right)/\left(n-k\right)$ for Kruskal–Wallis, and Cohen’s $d_{z}$ for within-participant contrasts, with 95% participant-bootstrap confidence intervals where applicable.

Multiplicity was controlled separately within each family of tests. Trial-level Kruskal–Wallis and participant-level Wilcoxon tests across the 117 informative features used both Bonferroni and Benjamini–Hochberg corrections. Cross-dataset marker tests across the 78 shared informative features used Benjamini–Hochberg correction within each dataset and target. The four personalization comparisons and the four primary participant-level protocol contrasts used Holm correction. Model-family, calibration, fusion, and ablation analyses were treated descriptively rather than through pairwise significance testing. Participant-bootstrap confidence intervals are reported for the model-family and fusion comparisons, and endpoint intervals for the calibration sweep are given in Table A7.

XGBoost gain importance was compared with permutation importance on a participant-independent held-out split using AUC scoring and with Shapley values obtained from a model-agnostic permutation explainer. The latter was applied to a 250-trial subsample using a 100-trial background. Feature-selection stability was assessed for top-*K* sets with K=10 and K=20 across the ten participant-independent folds using the Kuncheva consistency index [43] and mean pairwise Jaccard overlap. The Kuncheva index is chance-corrected, with 0 corresponding to the expected value under random selection.

To quantify participant-specific structure, we compared the EEG model with an identity-only predictor that assigns each test trial its participant’s positive-label rate estimated from the training data. The two predictors were evaluated on identical folds and rows under both epoch pooling and trial grouping. Participant identity was also decoded directly from trial-level features using random forest, with 32 classes for DEAP and 23 for DREAMER. All preprocessing, feature-extraction, evaluation, statistical analysis, and figure-generation code is included in the reproducibility package.

## 4. Results

We first quantify how the evaluation protocol shapes apparent performance and then report participant-independent recognition, the contribution of participant identity, within- and between-participant reliability, personalized modeling, and feature-level effect sizes, ablations and importance. Table 4 states which protocol each result assumes, and Table 5 consolidates the main AUCs with their configuration. Two estimands are kept distinct throughout: the pooled out-of-fold AUC, which ranks all trials of all participants together and is what conventional protocols report, and the mean per-participant AUC, which evaluates discrimination inside participant-specific evaluation units. Because the pooled estimand is also sensitive to between-participant differences in score level, the two quantify different components of optimistic performance, and neither supersedes the other.

**Table 5:** Headline AUCs and the configuration each assumes, ordered from the most permissive to the most conservative protocol. Leakage-prone rows are shown for reference only. The participant-independent and within-participant rows carry the conclusions.

Dataset	Label	Split	Normalization	Unit	Model	Valence	Arousal
DREAMER	fixed	epoch pooled (leakage-prone)	train-fold	epoch	XGBoost	0.755	0.784
DEAP	fixed	epoch pooled (leakage-prone)	train-fold	epoch	XGBoost	0.689	0.711
DEAP	fixed	trial-grouped, participants shared (leakage-prone)	train-fold	epoch	XGBoost	0.625	0.653
DEAP	fixed	within participants (personalized)	train-fold	trial	Logistic reg.	0.652	0.590
DEAP	particip. median	participant-independent (LOPO), transductive	per-particip.	trial	five classifiers	0.48–0.60	0.53–0.57
DEAP	fixed	participant-independent (GroupKFold), transductive	per-particip.	trial	XGBoost	0.535	0.543
DEAP	fixed	participant-independent (GroupKFold), inductive	train-fold	trial	XGBoost	0.493	0.447

### 4.1. Effect of the Evaluation Protocol

On DEAP, the fixed midpoint yields reasonably balanced classes, with high-class rates of 0.55 for valence and 0.58 for arousal, so class imbalance is mild. Under this absolute target, the participant-independent AUCs, obtained with GroupKFold and transductive per-participant standardization, are 0.535 for valence and 0.543 for arousal (Table 3). The per-participant median scheme, which we report as a within-person relative-state target rather than an absolute-affect target, gives 0.559 and 0.571. Across all four schemes the participant-independent AUCs span 0.535 to 0.559 for valence and 0.542 to 0.575 for arousal, so the qualitative conclusion does not depend on the binarization; this comparison was run on DEAP with XGBoost under GroupKFold and is specific to that combination.

**Figure 3:**
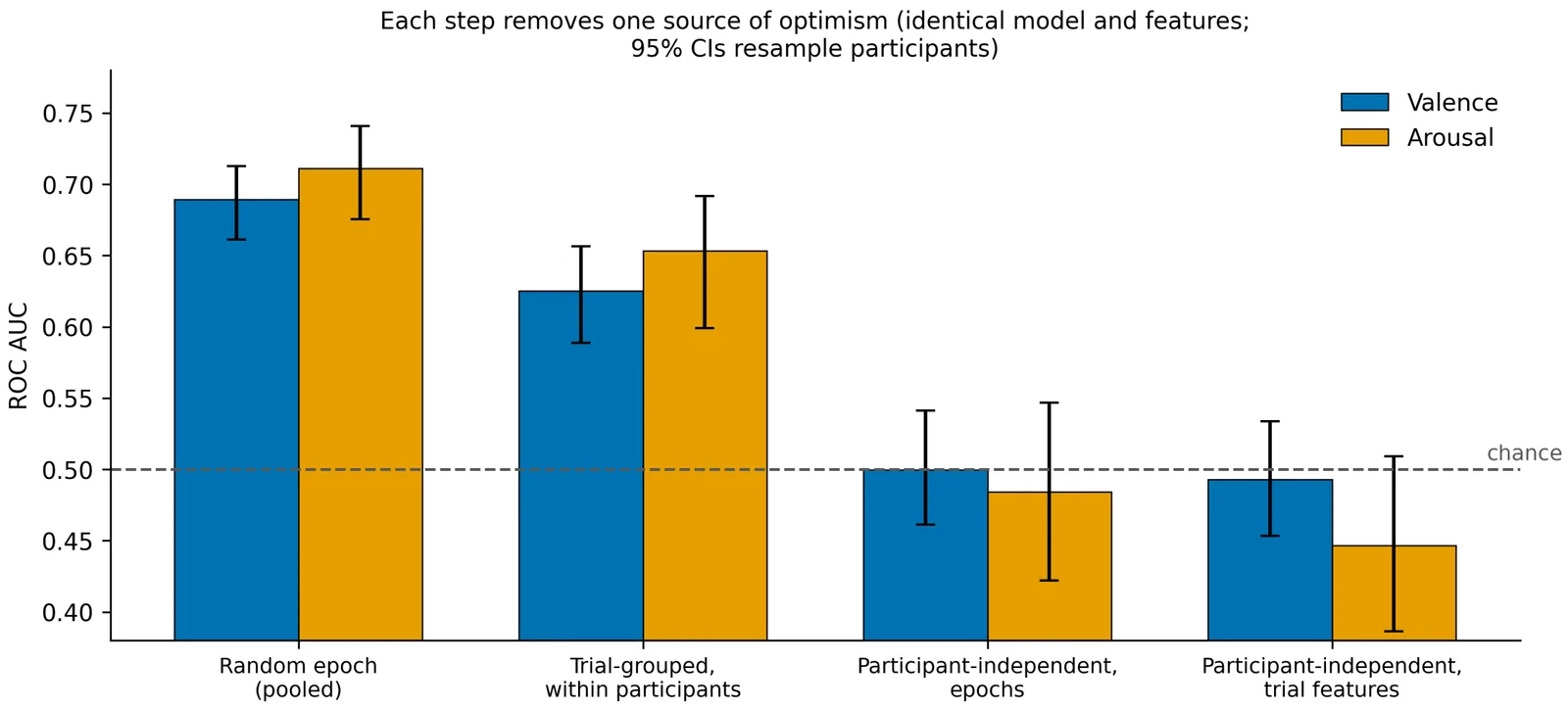
Apparent performance on DEAP depends on the evaluation protocol rather than the model.

Table 6 and Figure 3 decompose the optimism into distinct sources by changing one factor at a time while holding the model, feature definitions, labels, and training-fold standardization fixed. The first transitions alter the split structure, while the final transition changes the evaluation unit from epochs to trial-level features. Pooling overlapping epochs at random gives ROC-AUCs of 0.689 for valence and 0.711 for arousal. Grouping by trial, so that no epoch of a trial is split across the train/test boundary while the same participants still appear on both sides, removes near-duplicate-epoch leakage and lowers the AUCs to 0.625 and 0.653. Additionally separating participants brings performance to chance, 0.500 and 0.484 at the epoch level and 0.493 and 0.447 once features are aggregated to the trial, with every participant-independent interval spanning 0.50. Of the total 0.196 for valence and 0.265 for arousal, near-duplicate epochs therefore account for about 0.06 and participant separation for 0.125 and 0.169, while the change of evaluation unit contributes 0.007 for valence and 0.038 for arousal. Under this pooled estimand, participant overlap is thus the larger of the two channels on DEAP, though both are material. A comparison that removes both at once, as the pooled-versus-participant-independent contrast does, cannot by itself attribute the loss to either.

**Table 6:** Decomposition of the optimistic bias on DEAP under the pooled out-of-fold metric. Model, feature definitions, labels, and training-fold standardization are fixed; the rows progressively remove trial overlap and participant overlap, and the final row changes the evaluation unit to trial-level features. XGBoost, fixed-midpoint labels, and 117 informative features. Brackets are 95% bootstrap confidence intervals resampling participants.

Split	Same Trial	Same	Valence AUC	Arousal AUC
	Training/Test?	Participant?	[95% CI]	[95% CI]
Random epoch (pooled)	yes	yes	0.689 [0.661, 0.713]	0.711 [0.676, 0.741]
Trial-grouped, within participants	no	yes	0.625 [0.589, 0.656]	0.653 [0.599, 0.692]
Participant-independent, epochs	no	no	0.500 [0.461, 0.541]	0.484 [0.422, 0.547]
Participant-independent, trial features	no	no	0.493 [0.453, 0.534]	0.447 [0.386, 0.509]

The same decomposition was also evaluated at the participant level, with contrasts paired across the same 32 participants (Table 7). Grouping epochs by trial reduced the mean per-participant AUC by 0.091 for valence and by 0.127 for arousal, both significant after Holm correction, whereas the additional effect of separating participants was smaller and did not remain significant after correction. This differs from the pooled decomposition because pooled AUC is also sensitive to between-participant differences in score level, whereas per-participant AUC measures discrimination within participants. The small participant-level identity contrast is therefore not evidence that identity confounding is absent: the direct identity decoding, the variance decomposition and the matched identity-only baseline below all speak to it independently.

**Table 7:** Participant-level paired changes in AUC for the DEAP protocol decomposition. AUC was computed for each participant before and after each protocol change, and differences were paired within participants. Confidence intervals are unadjusted 95% intervals based on 10,000 participant-bootstrap resamples; *p*-values are from Wilcoxon signed-rank tests with Holm correction across the four contrasts.

Contrast	Target	Mean AUC Before	Mean AUC After	ΔAUC [95% CI]	Holm *p*
Overlapping epochs removed	Valence	0.651	0.560	−0.091 [−0.110, −0.076]	$1.9\times 10^{-9}$
Overlapping epochs removed	Arousal	0.622	0.494	−0.127 [−0.146, −0.111]	$1.9\times 10^{-9}$
Participants separated	Valence	0.560	0.522	−0.038 [−0.075, −0.002]	0.10
Participants separated	Arousal	0.494	0.515	+0.021 [−0.013, +0.052]	0.10

The participant-independent arousal AUC of 0.447 is not significantly different from chance (95% CI 0.386 to 0.509) and should therefore not be read as systematic below-chance prediction; per-participant standardization restores the same protocol to 0.543 (Table 3).

The same non-independence inflates significance. Features whose epoch-level Kruskal–Wallis *p* reaches $10^{-22}$ have trial-level effect sizes below 0.01 and participant-level *p*-values thirteen to seventeen orders of magnitude larger (Table A2 and Table A3).

**Figure 4:**
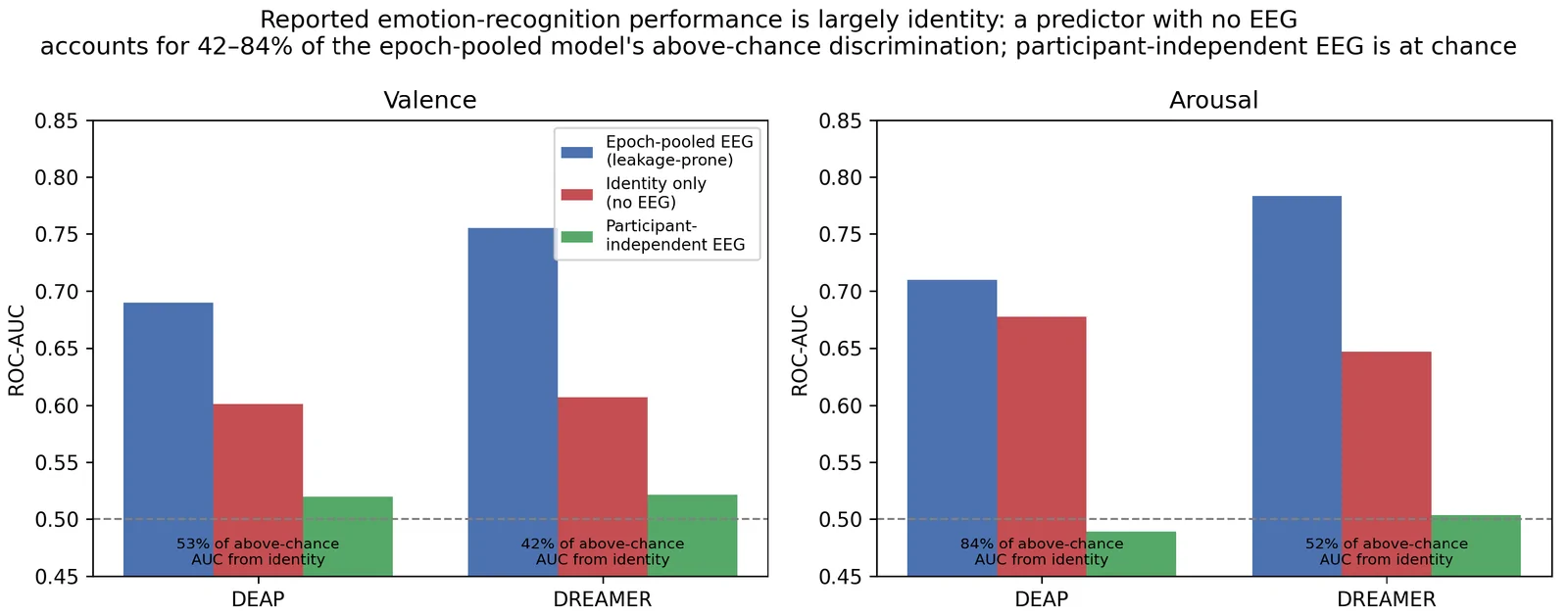
A predictor using no EEG accounts for 42–84% of the epoch-pooled model’s above-chance discrimination, while participant-independent EEG is at chance.

Much of this apparent performance is a function of participant identity rather than of emotion. Table 8 and Figure 4 quantify this on both datasets with the fixed-midpoint target. The EEG model and the identity-only predictor, which uses no EEG and labels every test trial with its participant’s training-set positive rate, are evaluated on identical folds and rows, so the two sides of the comparison are directly matched. Because ROC-AUC has a chance floor of 0.50, the informative comparison is against the discrimination achieved above chance. Under epoch pooling the identity-only predictor accounts for 53 percent of that discrimination for DEAP valence, 84 percent for DEAP arousal, 42 percent for DREAMER valence and 52 percent for DREAMER arousal. Under trial grouping, which leaves the participant prior available but prevents a test trial’s own epochs from informing it, the share is 46 percent for DEAP valence and rises to 94 percent for DEAP arousal, because trial grouping costs the EEG model more than it costs the participant prior. For DREAMER, the trial-grouped EEG model itself falls to chance, at 0.489 for valence and 0.551 for arousal with an interval spanning 0.50, so for valence, no share is defined, and for arousal, the figure of 48 percent carries that caveat. That collapse is informative: in DREAMER, almost all of the epoch-pooled performance is attributable to overlapping windows, the opposite ordering from DEAP, which fills a gap not resolved by the pooled decomposition. Centering features within each participant does not remove the effect, because the model re-identifies participants from residual structure and exploits their label prior. Only participant-independent evaluation removes it, at which point performance in this matched analysis falls to chance, between 0.489 and 0.521 across the four dataset–target combinations (Figure 4).

**Table 8:** An identity-only predictor that uses no EEG accounts for much of the EEG model’s above-chance discrimination. The EEG model and identity-only predictor are evaluated on identical folds and rows. Share is (identity−0.5)/(EEG−0.5). The EEG values are recomputed on the fold realization used for the matched identity-control comparison and therefore may differ slightly from the protocol decomposition values in Table 6. Under trial grouping, the participant prior remains available, but a test trial’s own epochs never enter training. Brackets are 95% bootstrap confidence intervals resampling participants.

Dataset	Target	EEG AUC [95% CI]	Identity-Only AUC [95% CI]	Share
Epoch-pooled evaluation				
DEAP	Valence	0.690 [0.663, 0.714]	0.601 [0.571, 0.623]	53%
DEAP	Arousal	0.710 [0.674, 0.740]	0.677 [0.628, 0.713]	84%
DREAMER	Valence	0.755 [0.708, 0.803]	0.607 [0.564, 0.640]	42%
DREAMER	Arousal	0.784 [0.743, 0.816]	0.647 [0.587, 0.684]	52%
Trial-grouped evaluation, participants still shared				
DEAP	Valence	0.621 [0.585, 0.653]	0.556 [0.509, 0.587]	46%
DEAP	Arousal	0.643 [0.588, 0.685]	0.635 [0.567, 0.680]	94%
DREAMER	Valence	0.489 [0.418, 0.560]	0.473 [0.383, 0.537]	not defined
DREAMER	Arousal	0.551 [0.487, 0.601]	0.524 [0.413, 0.590]	48% ^†^

^†^ The EEG model’s own interval spans chance here, so the share is reported with that caveat. For DREAMER valence under trial grouping, the EEG model is at chance, so there is no above-chance discrimination to apportion, and the ratio is not defined.

### 4.2. Participant-Independent Performance

Under leave-one-participant-out evaluation with transductive per-participant standardization and a Youden-tuned threshold, all model families perform modestly (Table 9). With the within-person relative-state target, the ROC-AUCs span 0.483 to 0.595 for valence and 0.532 to 0.566 for arousal, and apart from the linear support vector machine on valence, the confidence intervals of the families overlap, so the data do not identify one family as superior. Under the absolute fixed-midpoint target and the same protocol, the ranges are 0.506 to 0.579 and 0.438 to 0.554. Further optimization does not move this ceiling. A nested hyperparameter search, top-*k* feature selection and soft-voting ensembles reach at most 0.609 for valence and 0.587 for arousal across ten configurations (Table A4). Riemannian tangent-space classification with Euclidean Alignment gives 0.547 for valence and 0.502 for arousal on the compact montage and 0.547 and 0.557 on the full 32-channel montage. Because those single figures are the highest of a set selected on the reported metric, they are optimistic summaries rather than estimates of generalization. The limit therefore appears to lie in the participant specificity of the signal rather than in the choice of model, consistent with leakage-aware re-benchmarks of DEAP [11,46,47].

**Table 9:** Participant-independent classification. Leave-one-participant-out, per-participant median labels, transductive per-participant standardization, Youden-tuned threshold on an inner participant-aware split, trial-level unit, and 117 informative features. Brackets are 95% bootstrap confidence intervals resampling participants. Intervals overlap throughout for arousal; for valence, the linear support vector machine lies below the radial-basis-function machine and random forest.

Model	ROC-AUC [95% CI]	PR-AUC	Bal. Acc.	Recall_+_	Recall_−_
Valence					
Majority baseline	0.500 [0.500, 0.500]	0.495	0.500	0.00	1.00
Logistic Regression	0.532 [0.484, 0.580]	0.531	0.526	0.39	0.66
Linear SVM	0.483 [0.432, 0.539]	0.480	0.504	0.41	0.60
RBF SVM	0.594 [0.554, 0.633]	0.564	0.567	0.73	0.40
Random Forest	0.595 [0.559, 0.633]	0.557	0.580	0.57	0.59
XGBoost	0.567 [0.524, 0.610]	0.535	0.516	0.47	0.56
Arousal					
Majority baseline	0.500 [0.500, 0.500]	0.488	0.500	0.00	1.00
Logistic Regression	0.549 [0.513, 0.590]	0.545	0.524	0.43	0.62
Linear SVM	0.532 [0.497, 0.572]	0.532	0.527	0.50	0.56
RBF SVM	0.566 [0.529, 0.609]	0.543	0.535	0.59	0.48
Random Forest	0.565 [0.528, 0.600]	0.542	0.558	0.57	0.55
XGBoost	0.550 [0.513, 0.585]	0.537	0.524	0.59	0.46

### 4.3. A Participant-Specific Signature in the Feature Space

**Figure 5:**
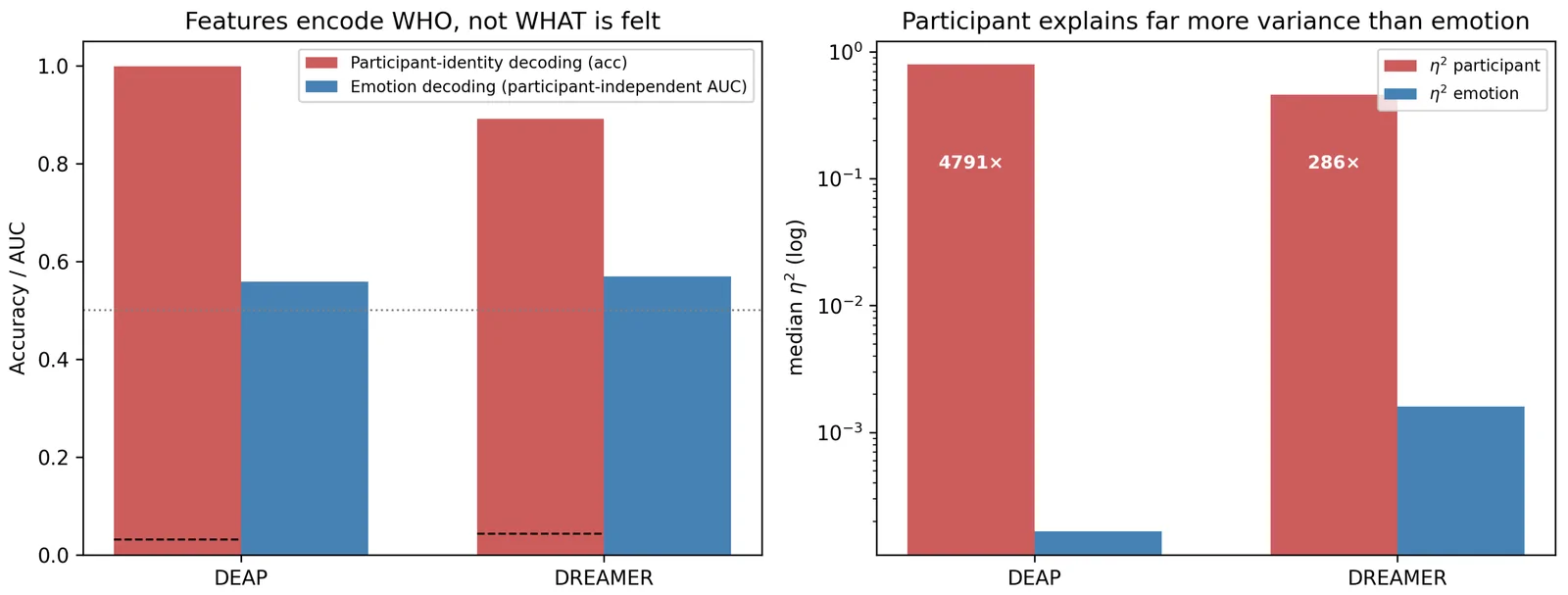
The features identify the person more than the emotion. Dashed line: chance.

The modest emotion performance does not mean that the features are uninformative; they are highly informative about the individual (Table 10 and Figure 5). A random forest decodes participant identity from the same trial-level features with accuracy of 0.998 on DEAP (chance of 0.031) and 0.891 on DREAMER (chance of 0.043), whereas participant-independent emotion decoding is near chance (about 0.55). A variance decomposition tells the same story: the median feature has an $\eta^{2}$ for participant of 0.79 against an $\eta^{2}$ for emotion of about 0.0002 on DEAP, so participant identity explains close to four orders of magnitude more variance than emotional state, with a ratio of about 290 on DREAMER. The qEEG descriptors proposed as affective markers are, first of all, participant-specific recording signatures. This signature conflates several stable and quasi-stable sources, including individual anatomy, electrode placement and impedance, recording-session conditions, and headset hardware; because both datasets provide essentially a single session per participant, we cannot separate a durable trait from session- and montage-specific factors, and we therefore describe it as a participant-specific recording signature rather than a cross-session biometric identity.

### 4.4. Within- and Between-Participant Reliability

**Figure 6:**
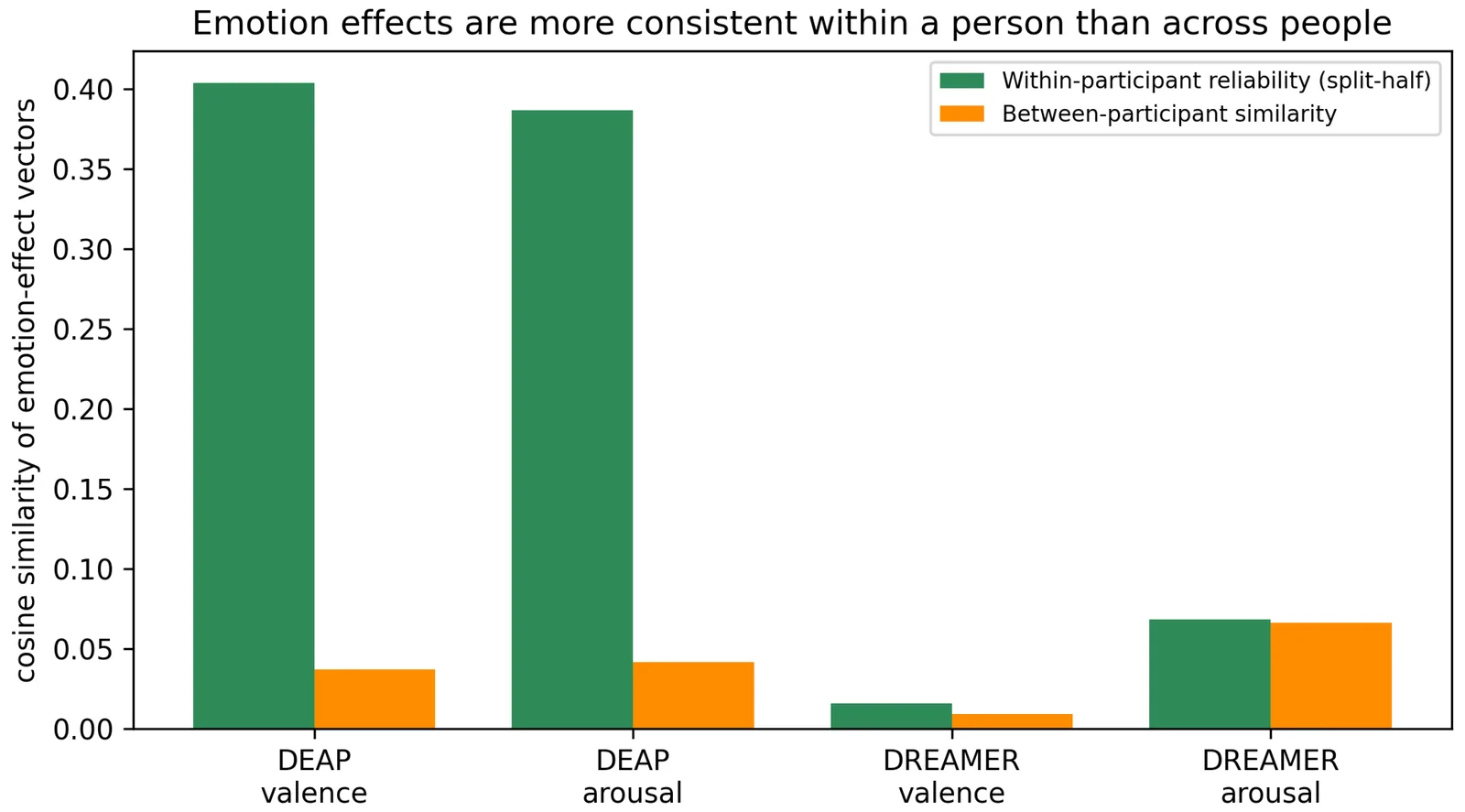
On DEAP, within-participant reliability of the emotion-effect vector far exceeds between-participant similarity; on DREAMER both are near zero and indistinguishable.

Within a participant, the emotion-effect vector, that is, the standardized high-minus-low contrast across all features, is reproducible on DEAP, with a split-half cosine similarity of 0.403 for valence and 0.387 for arousal. Across participants the same vectors are far less similar, at 0.037 and 0.042, and the per-feature effect direction opposes the majority for about 40 percent of features (Table 11 and Figure 6). On DREAMER both quantities are near zero and indistinguishable from each other. The within-participant signal on DEAP is therefore real, but its expression is largely individual.

The clearest test is cross-dataset replication on the four channels shared by the two datasets. Of the 78 informative shared features, three are FDR-significant within participants in DEAP for valence, and none are in DREAMER, while for arousal, none are significant in DEAP, and seven are in DREAMER, so no feature is jointly significant for either target (Table A5). The agreement between datasets is nevertheless dimension-dependent, and for arousal, the two summaries point in different directions in a way that requires explanation: For valence, the per-feature within-participant effect sizes are uncorrelated across datasets, with a Pearson *r* of −0.02 (95% CI −0.24 to 0.20), and their sign agrees on 40 percent of features (31 of 78, binomial p=0.09), no better than chance. For arousal, the effect sizes correlate moderately and reliably, with r=0.43 (95% CI 0.23 to 0.59, p<0.001) and sign agreement of 67 percent (52 of 78, p=0.004).

The absence of any jointly significant arousal feature does not contradict that correlation for three reasons. First, the joint criterion cannot be satisfied in the shared-channel analysis because none of the 78 shared DEAP arousal features survive FDR correction, so no feature can be significant in both datasets regardless of what DREAMER shows. Second, an important reason is channel coverage rather than absence of effect: the DEAP arousal features that do survive correction are parietal, at P3 (Table A3), and DREAMER does not record that site, so the shared-channel analysis excludes exactly the features carrying the strongest DEAP arousal effects. Third, the two summaries differ in what they test: a per-feature paired test corrected across 78 features evaluates each feature individually, whereas the correlation assesses concordance across the overall effect-size pattern. Taken together, the moderate correlation and significant sign agreement support cross-dataset consistency in the distributed arousal-related effect pattern across the shared frontal channels while providing no evidence for a single reproducible cross-dataset feature. The valence result provides no evidence of a shared pattern. Cross-dataset transfer of trained models remains close to chance for both targets (Table A6).

### 4.5. Personalized Models and Multimodal Fusion

Where the emotion-effect vector is reproducible within participants, modeling the participant helps; where it is not, it does not. The comparison in Table 12 is matched by design: a single fixed estimator is used in both arms, with identical features, identical trial-level representation, identical target definition and no threshold tuning, and one AUC is computed per participant in each arm so that the difference can be taken within participant. On DEAP, training on a participant’s own trials raises the mean per-participant valence AUC from 0.560 to 0.652. The paired difference is +0.092, with a 95% confidence interval of 0.029 to 0.157, a Wilcoxon *p* of 0.0105 and a Holm-adjusted *p* of 0.042 across the four tests of the table; the personalized model is better for 59 percent of participants. None of the other three targets show a reliable effect: DEAP arousal gives +0.056 (95% CI −0.009 to 0.119, Holm-adjusted p=0.31), DREAMER valence −0.027 (95% CI −0.115 to 0.070, Holm-adjusted p=0.41) and DREAMER arousal −0.032 (95% CI −0.088 to 0.025, Holm-adjusted p=0.41). The same pattern holds under the within-person relative-state target, where the DEAP valence difference is +0.112 (95% CI 0.045 to 0.177), and when both arms are evaluated under strictly inductive preprocessing. Personalization is therefore not a universal remedy: it helps for the one target where a reproducible within-participant effect was demonstrated, and DREAMER, where within-participant reliability is near zero (Table 11), shows no benefit.

**Table 12:** Matched personalized versus universal modeling. A single fixed estimator (logistic regression, C=1, balanced class weights) is used in both arms with identical features, trial-level representation and target definition, and no threshold tuning; only the training protocol differs. One ROC-AUC per participant per arm, paired within participant. Fixed-midpoint labels. Holm correction over the four tests shown; confidence intervals are 10,000 participant-bootstrap resamples.

Dataset	Target	*n*	Universal	Personalized	ΔAUC	95% CI	*p*	Holm *p*	% Better
DEAP	Valence	32	0.560	0.652	+0.092	[+0.029, +0.157]	0.0105	0.042	59
DEAP	Arousal	32	0.534	0.590	+0.056	[−0.009, +0.119]	0.102	0.307	66
DREAMER	Valence	23	0.556	0.529	−0.027	[−0.115, +0.070]	0.360	0.414	39
DREAMER	Arousal	23	0.553	0.521	−0.032	[−0.088, +0.025]	0.207	0.414	39

**Figure 7:**
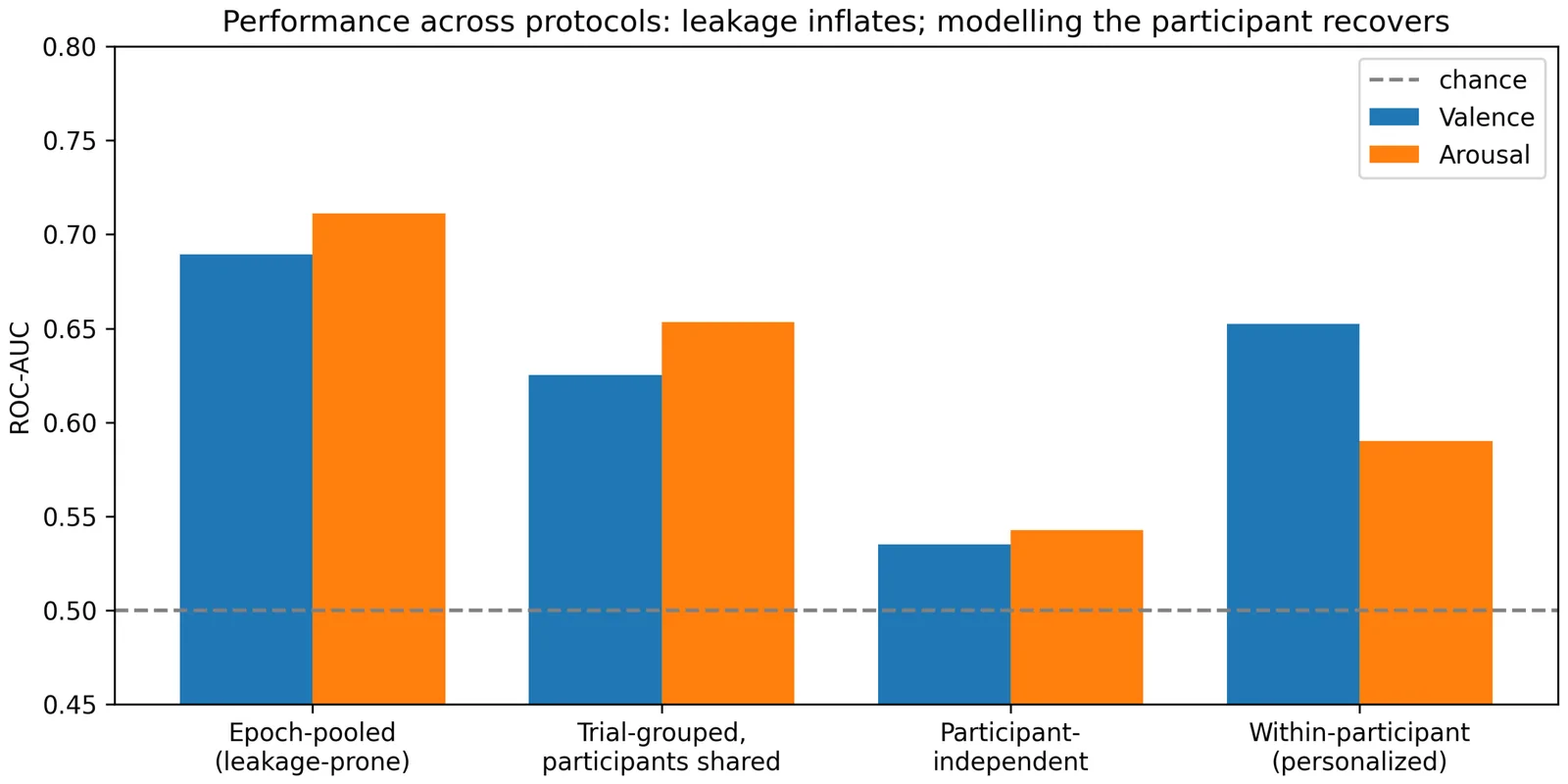
Performance across protocols, from epoch-pooled to participant-independent to personalized.

**Figure 8:**
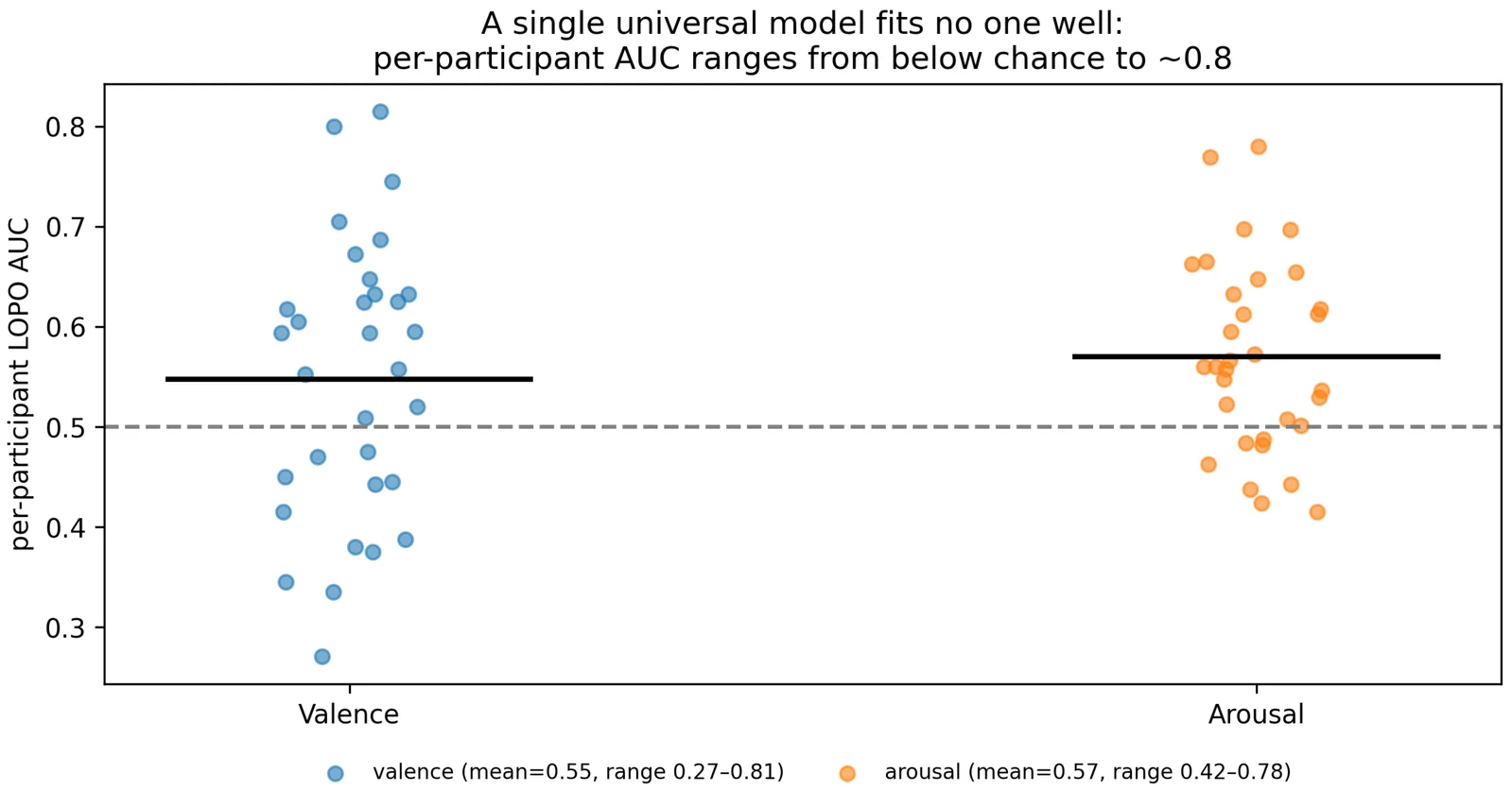
Per-participant leave-one-participant-out AUC ranges from below chance to about 0.8, so a single universal model does not fit the population well.

Adding a small number of labeled calibration trials from the target participant to a participant-independent model produces only marginal change up to k=20, a gain of 0.03 to 0.05 AUC whose confidence intervals overlap the uncalibrated model (Table A7). Training on a participant’s own data thus helps where a within-participant effect exists, whereas augmenting a universal model with a handful of target trials does not close the gap, which suggests that effective personalization needs a dedicated adaptation strategy or more extensive calibration than we tested (Figure 7). The wide spread of per-participant AUCs, from below chance to about 0.8 (Figure 8), is consistent with no single universal decision boundary fitting the population.

A natural hypothesis is that cortical EEG is the wrong signal and that peripheral autonomic measures would transfer better. This is not what we observe. Adding interpretable peripheral features, namely, heart-rate variability from blood-volume pulse, electrodermal activity, respiration, EMG, and temperature, to the EEG yields no reliable participant-independent gain (Table A8). For valence the fused model reaches 0.587 against 0.595 for EEG alone and for arousal 0.575 against 0.565, so the difference falls within the width of the confidence intervals in both directions; the peripheral features alone are weak, at 0.540 and 0.528. Because these comparisons carry no paired significance test, they indicate the absence of a detectable difference rather than demonstrated equivalence. The pattern of reliability within participants and idiosyncrasy across them is therefore a property of the affective signal in these datasets rather than of the EEG modality alone.

### 4.6. Effect Sizes, Ablations, and Feature Importance

**Figure 9:**
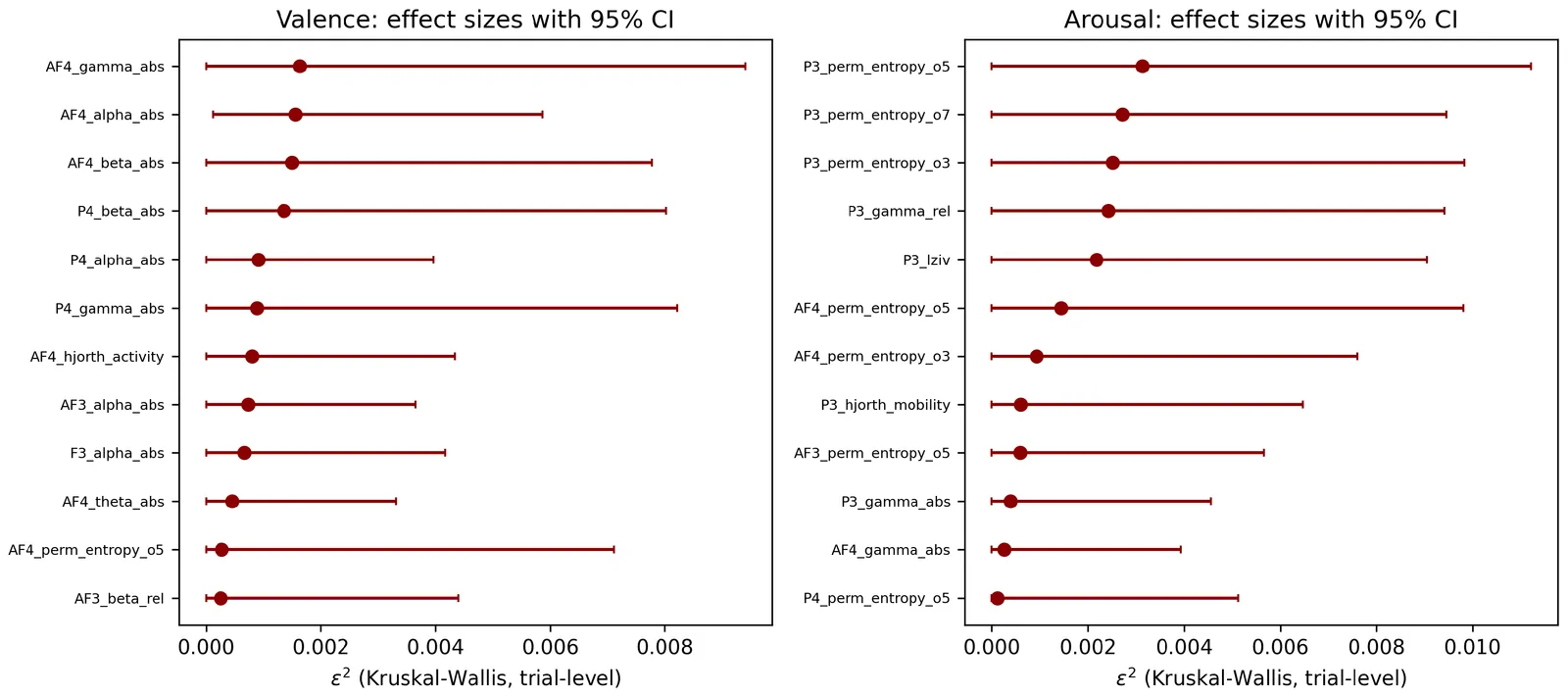
Trial-level $\epsilon^{2}$ effect sizes with 95% confidence intervals for the leading features.

Table A2 and Table A3 report, for the leading features, the trial-level $\epsilon^{2}$ with 95% bootstrap confidence intervals, the within-participant paired $d_{z}$, the FDR-corrected participant-level Wilcoxon *p*, and the inflated epoch-level *p* for contrast (Figure 9). For valence, the largest within-participant effects are in frontal and parietal alpha and theta power, with $d_{z}$ around 0.2 to 0.4, and four features reach FDR significance. For arousal, parietal permutation entropy and Lempel–Ziv complexity carry the strongest effects, with $d_{z}$ around 0.6, and six parietal features survive FDR correction, four complexity measures together with relative gamma and absolute beta power, all at P3. Frontal alpha asymmetry, emphasized in earlier analyses, is not significant under participant-level testing.

No feature family dominates classification (Table A9). Functional connectivity adds no measurable value, and connectivity alone is at chance, which answers the question of its incremental contribution. For valence the compact six-channel montage is as good as the full 32-channel set, and no single region dominates (Table A10); the differences between subsets are small relative to the confidence intervals of Table 9, so they are reported as an absence of a detectable difference rather than as equivalence.

Feature-selection stability is modest and is best read against its own chance level. The Kuncheva index is chance-corrected, so 0 is what random selection gives, and 1 indicates identical subsets; the observed values are 0.315 for valence and 0.290 for arousal at K=10, and 0.270 and 0.279 at K=20 (Table A11). In concrete terms, any two of the ten participant-independent folds share about 3.7 of their top-ten valence features and 3.5 of their top-ten arousal features, against 0.9 expected by chance. Selection is therefore well above chance but far from stable. The instability is confined to the tail rather than the whole ranking: one feature enters the top ten in every fold for each target, AF3 absolute alpha power for valence and P3 absolute gamma power for arousal, while most others appear in half the folds or fewer. Agreement between importance methods is likewise partial, with a Spearman correlation between XGBoost gain and permutation importance of 0.27 for valence and 0.39 for arousal (Table A12). Two caveats apply: gain and Shapley values come from a model fitted on all trials whereas permutation importance is measured on a participant-independent held-out split, so two different fitted models are compared, and the Shapley values are a subsampled model-agnostic approximation rather than the tree-specific estimator. Gain alone is accordingly an unreliable indicator of predictor relevance.

## 5. Discussion

We summarize the principal findings, draw out their implications for personalized affective computing, and set out the main limitations and ethical considerations.

### 5.1. Principal Findings

The aim of this study was to identify interpretable qEEG biomarkers of valence and arousal. The analyses show that this goal, as it is usually framed, rests on an evaluation design that does not test the property being claimed. A biomarker must be reproducible across the population to which it is applied, yet the evidence usually offered, namely, high accuracy under epoch-pooled cross-validation and very small *p*-values, is exactly what epoch-level leakage and pseudo-replication produce even when the underlying effect is weak and personal. Once both artifacts are removed, the picture changes: the markers are reproducible within a person on DEAP, near zero within a person on DREAMER, and idiosyncratic across people and datasets.

Three findings converge on this conclusion. Participant identity is highly decodable from the same features proposed as affective markers, while participant-independent emotion decoding is barely above chance. A variance decomposition attributes close to four orders of magnitude more feature variance on DEAP and between two and three on DREAMER to who the participant is rather than to what they feel. The direction of the emotion effect is largely not shared across participants, reversing for about 40 percent of features; arousal effect sizes show a moderate cross-dataset correlation while valence effects do not, and no single feature is separately established in both datasets. A single global decision boundary cannot fit the population, which is what the wide spread of per-participant performance reflects. This is consistent with long-standing observations of inter-individual EEG variability arising from anatomy, skull conductivity, electrode placement and trait differences, and it explains why every model family we tried, from regularized linear models to kernel machines, random forests, gradient boosting and Riemannian classifiers with Euclidean Alignment, saturates in the same narrow band above chance. The limiting factor is the mismatch between a universal target and a participant-specific signal, not the choice of model.

The pooled and participant-level decompositions emphasize different components of optimistic performance, because pooled AUC also reflects between-participant differences in score level; both nevertheless support the conclusion that conventional evaluation overstates transferable affective information.

Comparison with a peripheral modality is instructive here. Long et al. [45] report participant-invariant emotional information decoded from cardiac signals acquired by photonic sensing, which indicates that the ceiling observed here need not hold for every physiological channel. Our own peripheral features do not settle the question either way: the DEAP autonomic set includes cardiac-derived measures, reaches 0.540 for valence and 0.528 for arousal on its own under leave-one-participant-out, and adds no detectable participant-independent gain to the EEG (Table A8); DREAMER’s ECG was not analyzed here. Within these datasets, then, the limitation is a property of the affective signal as recorded rather than of the cortical modality alone.

### 5.2. Implications for Personalized Affective Computing

If the affective signal is participant-specific, the natural response is to model the participant. Under a matched comparison, a classifier trained on a single participant’s own data outperforms one trained on all other participants for DEAP valence and does not for DEAP arousal or for either DREAMER target. Adding a limited number of labeled calibration trials from the target participant to a universal model yields only modest gains. Personalization is therefore worth pursuing where a reproducible within-participant effect has been demonstrated, and it should not be assumed to help otherwise; effective adaptation appears to require a dedicated strategy rather than a handful of extra trials. This view reframes much of the transfer learning and foundation-model literature, including multi-source transfer [32], Euclidean Alignment [33], contrastive cross-dataset models [34], multi-dataset pre-training [35] and test-time adaptation [38], as different mechanisms for injecting participant- or domain-specific information so that a shared model can be specialized. Related work on few-shot multimodal affective representation learning [44] suggests a complementary route, in which the burden of generalization is carried by large pre-trained or multimodal representations rather than by a compact hand-crafted feature set. Our results give a feature-level reason why such adaptation is necessary rather than optional, and they suggest that interpretable markers are the most useful not as population norms but as per-participant references tracked over time, for example, against an individual’s own baseline, a mode already familiar in clinical qEEG.

The stability results limit how strongly individual qEEG features can be interpreted as affective markers. Relative to chance, feature selection is more stable than random: two participant-independent folds share about 3.7 of their top-10 features, compared with 0.9 expected by chance. However, the selected sets remain far from fully stable, and agreement across importance criteria is only partial. This makes strong claims about individual features difficult, since changes in the training fold, importance criterion, or feature pool can alter which features are selected. More consistent patterns emerge at the feature-family level. One feature per target is selected in every fold, while parietal complexity, and gamma and beta power show the most consistent associations with arousal within participants and frontal and parietal alpha and theta power with valence. We therefore interpret the instability as a limitation on feature-level attribution rather than evidence against a within-participant affective signal, whose reproducibility is supported independently by the split-half analysis. Feature-selection stability should therefore be reported alongside predictive performance when individual qEEG markers are interpreted.

These results also explain the gap to the reported state of the art. Many DEAP results above 90 percent are obtained with participants shared between training and test data, often with epoch pooling, and the audit here shows how much of the gap to honest participant-independent performance is attributable to these choices. Where independent groups have re-evaluated DEAP under matched, leakage-aware protocols, participant-independent accuracy clusters around 0.51 to 0.70 [11,46,47], in line with the present results. We therefore compare only within matched protocols, and Table 1 groups the literature accordingly. The features are not meaningless. Within participants, frontal and parietal alpha and theta power show the most reliable association with valence, while parietal complexity measures and gamma power associate with arousal, with directions that are reproducible across split halves. The supported claim is therefore narrower: these are candidate within-participant state indicators rather than universal population biomarkers, and the frontal alpha asymmetry emphasized in earlier work does not survive participant-level testing.

### 5.3. Practical Recommendations for Future DEAP and DREAMER Studies

The results suggest several practical choices that can make future studies easier to interpret and compare:

1. Describe the evaluation protocol clearly. Studies should report whether participants are shared between training and test data, whether epochs from the same trial can appear on both sides of the split, the unit used for evaluation, and how normalization or other fitted transformations are estimated.
2. Keep trials intact and separate participants for population-level claims. Overlapping epochs from the same trial should remain in the same split, and predictions should be aggregated at the trial level before scoring. If the goal is to generalize to unseen individuals, the training/test split should also be participant-independent.
3. Use participants as the statistical unit. Confidence intervals and statistical tests should reflect participant-level independence rather than treating epochs as independent observations. When pooled and per-participant performance are both reported, they should be interpreted separately because they capture different aspects of the evaluation.
4. State how labels are defined. Fixed-midpoint labels and per-participant median labels correspond to different tasks. The former supports an absolute or population-level interpretation, whereas the latter defines a within-person relative-state problem.
5. Check how much performance can be explained by participant identity. An identity-based control can be evaluated on the same folds and rows as the EEG model to estimate how much of the apparent discrimination is available from participant-specific label tendencies alone. The correction applied to each family of statistical tests should also be stated explicitly.
6. Report model and feature stability, not only peak performance. When several classifiers or feature sets are evaluated, reporting the range of results and the stability of selected features is more informative than focusing only on the single highest score. Releasing out-of-fold predictions also facilitates participant-level comparisons and independent re-analysis.

Applied to DEAP and DREAMER, these choices help explain why pooled results can differ substantially from participant-independent performance and make the contribution of participant-specific structure easier to identify.

### 5.4. Limitations and Ethical Considerations

The conclusions concern the qEEG feature families studied here. Sample entropy and Higuchi fractal dimension were part of the extraction specification but returned degenerate constant values and are excluded, so the analyzed set comprises 117 features on the compact montage; their contribution is untested rather than shown to be absent. Although Riemannian covariance models did not exceed the ceiling, richer or end-to-end deep representations could in principle extract more transferable structure, and testing this is worthwhile future work. The two datasets differ in montage (the 14-channel DREAMER headset lacks the parietal sites that carried the strongest DEAP arousal effects, which is why the cross-dataset marker analysis is restricted to four frontal channels), stimuli, and population, so cross-dataset non-replication mixes genuine participant specificity with acquisition differences; this is a realistic deployment condition, but it limits mechanistic interpretation. The labels are binary, and the emotions are laboratory-induced. Per-participant standardization and personalized models assume access to data from the target individual, which suits calibrated or clinical use but not zero-shot deployment. Finally, the ease with which a participant is re-identified from these features, at about 99 percent within a single session, is a reminder that qEEG descriptors act as quasi-identifiers. Whether this signature is stable enough across days and recording setups to constitute a true biometric is not established by single-session data, but the within-session result already gives affective EEG systems biometric and privacy implications, and they should be designed accordingly. Personalized models also require collecting and storing per-user calibration data, with the associated consent and data-governance obligations. The reported performance reflects a small, demographically narrow, laboratory sample and should not be assumed to hold for clinical populations, naturalistic emotion or consumer hardware without dedicated validation.

## 6. Conclusions

A leakage-free re-evaluation on two datasets shows that emotion-related effects in interpretable qEEG markers of valence and arousal are reproducible within participants on DEAP but considerably weaker on DREAMER and that neither dataset gives convincing evidence of markers that generalize across individuals or datasets. Leakage-prone evaluation inflates both classification performance, by 0.196 for valence and 0.265 for arousal in pooled AUC on DEAP, and statistical significance, by more than ten orders of magnitude in the most extreme cases. Under the pooled metric, participant overlap is the larger of the two contributing channels, at 0.125 for valence and 0.169 for arousal, with overlapping epochs contributing about 0.06; a participant-level paired analysis emphasizes the epoch contribution instead. Once these artifacts are removed, the same features that decode participant identity with very high accuracy decode emotional state only marginally above chance, and a predictor using no EEG accounts for 42 to 84 percent of the epoch-pooled model’s above-chance discrimination from identity alone. The emotion effect is reproducible within a person on DEAP but heterogeneous across people, its direction reverses for about 40 percent of features, and no feature is separately established in both datasets, although arousal effect sizes do correlate moderately across the shared channels. Consistent with a participant-specific signal, a matched comparison shows that training on a participant’s own data helps for DEAP valence and not for the other three targets, every model family and an additional modality saturate in the same narrow band, and further optimization does not change that. Feature selection is well above chance but far from stable, which limits attribution to individual markers even where family-level associations hold. These results argue that robust affective EEG should be pursued through personalized and calibrated models and that interpretable qEEG indicators are best used as per-participant references rather than universal norms. To support this aim we release a fully reproducible pipeline with effect sizes, confidence intervals, stability and importance analyses, ablations and matched evaluation protocols. Future work should quantify how many per-participant data personalization requires, evaluate richer and multimodal representations under the same leakage-free protocols, and integrate interpretable per-participant markers as priors within adaptive models.

## Figures and Tables

**Table 1:** Representative DEAP studies grouped by whether participants are shared between training and test data. Reported metrics, evaluation units, and normalization strategies differ across studies and should therefore be considered when comparing results. “None” indicates that no additional normalization step was reported beyond the stated preprocessing. For the present work, the range across the five classifiers under the fixed-midpoint target is shown; Section 4 reports the same classifiers under the per-participant median scheme.

Study	Features	Classifier	Metric	Valence	Arousal	Eval. Unit	Normaliz.
Participants shared between training and test data							
Koelstra et al. [9]	Spectral PSD	Gaussian NB	Acc	0.576	0.620	Trial	None (log spectral power only)
Atkinson & Campos [12]	Band power (mRMR)	SVM	Acc	0.731	0.731	Trial	None on signal; per-particip. feature discretization
Alhagry et al. [20]	Raw EEG	LSTM	Acc	0.854	0.857	Trial	None (raw EEG)
Yang et al. [21]	Raw EEG	CNN-RNN	Acc	0.908	0.910	1 s segment	Pre-trial baseline removal + z-score
Hasan & Islam [16]	Temporal (DE, HFD)	XGBoost	Acc	0.89	0.88	3 s segment	None (baseline discarded)
Participants disjoint between training and test data							
Wang et al. [46]	Raw EEG	Self-superv.	Acc	0.655	0.697	4 s segment	None (resampling only)
Tang et al. [47]	Spatio-temporal	Deep net	Acc	0.675	0.683	6 s segment, 3 s overlap	In-network instance and batch norm.
Kukhilava et al. [11]	Re-benchmark	Various	Acc	0.51–0.53	0.53–0.57	4 s segment	None (baseline cropped)
This work	Spectral + nonlinear qEEG	RF/LR/SVM/ XGB	AUC	0.51–0.58	0.44–0.55	Trial	Per-particip. (transductive)

**Table 2:** Summary of the two datasets.

Characteristic	DEAP	DREAMER
Participants	32	23
Stimuli/trials	40 music videos	18 film clips
EEG channels	32 (10–20)	14 (Emotiv EPOC)
Sampling rate	128 Hz	128 Hz
Other signals	8 peripheral	ECG
Rating scale	1–9	1–5

**Table 3:** Sensitivity to the label binarization scheme. XGBoost, participant-independent GroupKFold (ten folds), trial-level unit, transductive per-participant standardization, and 117 informative features. Brackets are 95% bootstrap confidence intervals resampling participants.

Labeling Scheme	Valence			Arousal		
	Balance	N	AUC [95% CI]	Balance	N	AUC [95% CI]
Fixed midpoint (>5)	0.55	1280	0.535 [0.489, 0.580]	0.58	1280	0.543 [0.507, 0.579]
Global median	0.50	1280	0.539 [0.494, 0.585]	0.50	1280	0.542 [0.510, 0.575]
Per-participant median	0.49	1280	0.559 [0.520, 0.599]	0.49	1280	0.571 [0.539, 0.607]
Margin (|r−5|≥2)	0.63	595	0.536 [0.483, 0.601]	0.57	523	0.575 [0.521, 0.629]

**Table 10:** The qEEG features encode participant identity far more than emotional state. Identity is decoded with random forest from trial-level features; emotion AUC is participant-independent logistic regression. Variance components are median $\eta^{2}$ across the informative features. Emotion columns refer to valence.

Dataset	Identity Acc.	Identity Chance	Emotion AUC	$\eta_{\mathrm{particip}.}^{2}$	$\eta_{\mathrm{emotion}}^{2}$	Ratio
DEAP	0.998	0.031	0.550	0.793	0.00017	4791×
DREAMER	0.891	0.043	0.564	0.458	0.00160	286×

**Table 11:** Within- versus between-participant consistency of the emotion-effect vector. Reliability is the split-half cosine similarity within a participant; between-participant similarity is the mean pairwise cosine; the sign-flip rate is the mean proportion of participants whose effect direction opposes the majority (0 = all agree, 0.5 = random).

Dataset/Target	Within-Particip. Reliability	Between-Particip. Similarity	Sign-Flip Rate
DEAP/valence	0.403	0.037	0.41
DEAP/arousal	0.387	0.042	0.40
DREAMER/valence	0.016	0.009	0.41
DREAMER/arousal	0.069	0.066	0.36

## Data Availability

DEAP is available from https://www.eecs.qmul.ac.uk/mmv/datasets/deap/ (doi:10.1109/T-AFFC.2011.15, accessed on 18 August 2026), and DREAMER from https://zenodo.org/records/546113 (accessed on 18 August 2026); both are third-party datasets obtained under their custodians’ own terms and are not redistributed here. All preprocessing, feature-extraction, evaluation, statistics and figure-generation code, and the exact hyperparameters reported here, and the computed results in machine-readable form are openly available at https://github.com/ema-pandilova/qeeg-emotion-pipeline (accessed on 18 August 2026), so that every number in this paper can be recomputed. The 32-channel feature table used for the montage and feature-family ablations is regenerated by the enhanced-pipeline configuration in that repository rather than by the compact-montage extraction scripts.

## Abbreviations

The following abbreviations are used in this manuscript:

AUC	Area under the receiver operating characteristic curve
CI	Confidence interval
DEAP	Database for Emotion Analysis using Physiological signals
DREAMER	Database for emotion recognition through EEG and ECG signals
EEG	Electroencephalography
FAA	Frontal alpha asymmetry
FDR	False discovery rate
LOPO	Leave-one-participant-out
qEEG	Quantitative electroencephalography
ROC	Receiver operating characteristic
SAM	Self-Assessment Manikin

## Appendix A. Additional Tables

These tables report the classifier configuration, feature-level effect sizes, ablations, stability and importance analyses, and the secondary label and transfer schemes that support the results in the main text.

**Table A1:** Feature-extraction hyperparameters, identical for DEAP and DREAMER. Sample entropy and Higuchi fractal dimension returned constant values for every epoch of both datasets and were excluded from all analyses; the analyzed set comprises 117 features on the DEAP six-channel montage and 78 on the channels shared with DREAMER.

Parameter	Value
Bandpass filter	0.5–40 Hz, fourth-order Butterworth (zero-phase)
Epoch length/overlap	4 s/2 s (50%)
Welch PSD	Hann window, nperseg = epoch, 50% segment overlap
Frequency bands	δ 1–4, θ 4–8, α 8–13, β 13–30, γ 30–40 Hz
Relative power denominator	total power 1–40 Hz
Permutation entropy	orders 3, 5, 7; delay 1; normalized
Lempel–Ziv complexity	median-threshold binarization, normalized
Hjorth parameters	activity, mobility, complexity
Frontal alpha asymmetry	$\ln P_{\alpha }^{\mathrm{right}}-\ln P_{\alpha }^{\mathrm{left}}$, pairs (F4, F3), (AF4, AF3), (P4, P3)
PLV/coherence (32-ch only)	per band, Hilbert phase, selected pairs
Sample entropy	m=2, r=0.2·SD—degenerate, excluded
Higuchi fractal dimension	$k_{\max}=10$—degenerate, excluded

**Table A2:** Leading valence features by the within-participant paired test on DEAP (per-participant median split, n=32, 117 features). Columns give the trial-level Kruskal–Wallis $\epsilon^{2}$ with 95% bootstrap CI over participants, the within-participant $d_{z}$, the raw and FDR-corrected participant-level Wilcoxon *p*, and the epoch-level *p* shown only to expose pseudo-replication.

Feature	$\epsilon^{2}$ [95% CI]	$d_{z}$	$p_{\mathrm{particip}.}$	FDR	$p_{\mathrm{KW},\mathrm{epoch}}$
F3_alpha_abs	0.0007 [0.0000, 0.0043]	+0.21	3.3 × 10^−4^	0.039	1.2 × 10^−7^
F3_theta_abs	0.0002 [0.0000, 0.0028]	+0.35	1.1 × 10^−3^	0.045	2.9 × 10^−6^
P4_alpha_abs	0.0009 [0.0000, 0.0046]	+0.28	1.5 × 10^−3^	0.045	1.1 × 10^−11^
F3_hjorth_activity	0.0000 [0.0000, 0.0022]	+0.15	1.5 × 10^−3^	0.045	1.3 × 10^−5^
AF3_alpha_abs	0.0007 [0.0000, 0.0041]	+0.05	4.7 × 10^−3^	0.105	1.1 × 10^−8^
AF4_alpha_abs	0.0016 [0.0001, 0.0055]	+0.17	5.4 × 10^−3^	0.105	3.5 × 10^−12^
AF3_theta_abs	0.0000 [0.0000, 0.0024]	+0.19	6.5 × 10^−3^	0.108	7.6 × 10^−6^
AF3_beta_rel	0.0003 [0.0000, 0.0042]	−0.37	8.3 × 10^−3^	0.121	6.0 × 10^−7^
P4_hjorth_activity	0.0002 [0.0000, 0.0028]	+0.23	2.1 × 10^−2^	0.253	2.1 × 10^−7^
P4_theta_abs	0.0000 [0.0000, 0.0018]	+0.38	2.2 × 10^−2^	0.253	2.2 × 10^−5^

**Table A3:** Leading arousal features by the within-participant paired test on DEAP (columns as in Table A2); six features at P3 survive FDR correction, four complexity measures together with relative gamma and absolute beta power.

Feature	$\epsilon^{2}$ [95% CI]	$d_{z}$	$p_{\mathrm{particip}.}$	FDR	$p_{\mathrm{KW},\mathrm{epoch}}$
P3_perm_entropy_o5	0.0031 [0.0000, 0.0113]	+0.62	2.8 × 10^−5^	0.002	2.3 × 10^−22^
P3_perm_entropy_o7	0.0027 [0.0000, 0.0085]	+0.64	3.9 × 10^−5^	0.002	2.8 × 10^−20^
P3_perm_entropy_o3	0.0025 [0.0000, 0.0096]	+0.61	6.7 × 10^−5^	0.003	9.4 × 10^−21^
P3_gamma_rel	0.0024 [0.0000, 0.0098]	+0.46	3.3 × 10^−4^	0.009	7.4 × 10^−18^
P3_lziv	0.0022 [0.0000, 0.0088]	+0.63	3.6 × 10^−4^	0.009	1.7 × 10^−15^
P3_beta_abs	0.0000 [0.0000, 0.0016]	+0.45	1.8 × 10^−3^	0.035	2.3 × 10^−2^
P3_gamma_abs	0.0004 [0.0000, 0.0044]	+0.28	6.9 × 10^−3^	0.111	8.2 × 10^−10^
F4_perm_entropy_o7	0.0000 [0.0000, 0.0025]	+0.42	9.3 × 10^−3^	0.111	9.9 × 10^−3^
F4_perm_entropy_o5	0.0000 [0.0000, 0.0032]	+0.44	1.1 × 10^−2^	0.111	1.0 × 10^−4^
F4_perm_entropy_o3	0.0000 [0.0000, 0.0029]	+0.45	1.1 × 10^−2^	0.111	3.6 × 10^−4^

**Table A4:** Participant-independent performance after extensive optimization on DEAP (per-participant median labels, transductive standardization, GroupKFold, mean of per-participant AUCs). Ten configurations were evaluated per target. Because the selection is made on the reported metric, the highest value is an optimistic summary.

Target	Range over 10 Configurations	Highest Value [95% CI]	Configuration
Valence	0.544–0.609	0.609 [0.568, 0.648]	6-channel, tuned random forest
Arousal	0.535–0.587	0.587 [0.553, 0.622]	6-channel, tuned random forest

**Table A5:** Cross-dataset within-participant marker replication on the four channels shared by DEAP and DREAMER. Counts are over the 78 informative shared features; a feature counts as replicated if it is FDR-significant in both datasets with the same effect direction. Sign agreement carries a two-sided binomial *p* against chance.

Target	DEAP FDR-sig	DREAMER FDR-sig	Replicated	Sign Agreement	Binomial *p*
Valence	3/78	0/78	0	0.40 (31/78)	0.089
Arousal	0/78	7/78	0	0.67 (52/78)	0.004

**Table A6:** Within-dataset and cross-dataset transfer (→) AUC on the four channels shared by DEAP and DREAMER (78 informative features, per-participant median labels, and transductive per-participant standardization). Within-dataset columns are participant-independent; transfer columns train on one dataset and test on the other.

Target (Model)	DEAP	DREAMER	DEAP→DREAMER	DREAMER→DEAP
Valence (logistic reg.)	0.589	0.552	0.498	0.470
Valence (XGBoost)	0.568	0.571	0.510	0.534
Arousal (logistic reg.)	0.542	0.561	0.526	0.503
Arousal (XGBoost)	0.530	0.545	0.555	0.559

**Table A7:** Participant-adaptive calibration on DEAP: leave-one-participant-out ROC-AUC as *k* labeled trials from the target participant are added to the universal training set (per-participant median labels, mean of per-participant AUCs). At the endpoints, the 95% participant-bootstrap intervals are 0.548 [0.499, 0.593] at k=0 and 0.596 [0.551, 0.641] at k=20 for valence with logistic regression, 0.605 [0.569, 0.643] and 0.643 [0.607, 0.676] for valence with random forest, 0.570 [0.539, 0.602] and 0.608 [0.576, 0.642] for arousal with logistic regression, and 0.575 [0.543, 0.608] and 0.601 [0.567, 0.634] for arousal with random forest; every k=20 interval overlaps its k=0 interval.

Target (Model)	*k* = 0	*k* = 4	*k* = 8	*k* = 12	*k* = 16	*k* = 20
Valence (logistic reg.)	0.548	0.560	0.567	0.575	0.586	0.596
Valence (random forest)	0.605	0.605	0.619	0.628	0.623	0.643
Arousal (logistic reg.)	0.570	0.580	0.585	0.594	0.596	0.608
Arousal (random forest)	0.575	0.584	0.585	0.592	0.606	0.601

**Table A8:** Adding peripheral autonomic features to the EEG does not improve participant-independent performance on DEAP (leave-one-participant-out, random forest, per-participant median labels, and trial-level unit). Differences between EEG-only and fused models are within the width of their confidence intervals in both directions.

Target	EEG Only [95% CI]	Peripheral Only [95% CI]	Fused [95% CI]
Valence	0.595 [0.559, 0.633]	0.540 [0.502, 0.581]	0.587 [0.551, 0.626]
Arousal	0.565 [0.528, 0.600]	0.528 [0.490, 0.567]	0.575 [0.542, 0.610]

**Table A9:** Feature-family ablation on DEAP (XGBoost, participant-independent GroupKFold, per-participant median labels, transductive per-participant standardization, and trial-level unit). Counts are informative features. Functional connectivity adds no measurable value.

Feature Family	6-Channel Set			32-Channel Set		
	#	Val. AUC	Aro. AUC	#	Val. AUC	Aro. AUC
Spectral only	75	0.564	0.554	500	0.574	0.550
Nonlinear only	42	0.531	0.543	96	0.527	0.536
All features	117	0.559	0.571	686	0.569	0.556
Connectivity only	–	–	–	90	0.516	0.545
All minus connectivity	–	–	–	596	0.565	0.551

**Table A10:** Channel ablation on DEAP (configuration as in Table A9). Counts are informative features. The compact six-channel montage performs comparably to the full 32-channel set, and no single region dominates.

Channel Subset	Valence		Arousal	
	#	AUC	#	AUC
Frontal (F3, F4, AF3, and AF4)	78	0.564	78	0.531
Parietal (P3 and P4)	39	0.553	39	0.556
All 6 channels	117	0.559	117	0.571
All 32 channels	686	0.569	686	0.556

**Table A11:** Feature-selection stability across the ten participant-independent folds on DEAP (XGBoost gain ranking and 117 informative features). The Kuncheva index is chance-corrected, so 0 is the value expected from random selection; the implied overlap column gives the mean number of features shared between any two folds.

Target	*K*	Kuncheva	Jaccard	Implied Overlap	Chance Overlap
Valence	10	0.315	0.235	3.7/10	0.9/10
Valence	20	0.270	0.250	7.9/20	3.4/20
Arousal	10	0.290	0.217	3.5/10	0.9/10
Arousal	20	0.279	0.255	8.0/20	3.4/20

**Table A12:** Agreement between feature-importance methods on DEAP (Spearman correlation over the 117 informative features and top-10 Jaccard overlap). Gain and Shapley values come from a model fitted on all trials; permutation importance is measured on a participant-independent held-out split, so two different fitted models are compared. Shapley values were obtained with a model-agnostic permutation explainer on a 250-trial subsample.

Target	$\rho_{\mathrm{gain},\mathrm{Shap}}$	$\rho_{\mathrm{gain},\mathrm{perm}}$	$\rho_{\mathrm{Shap},\mathrm{perm}}$	$J_{10}^{\mathrm{gain},\mathrm{Shap}}$	$J_{10}^{\mathrm{gain},\mathrm{perm}}$
Valence	0.42	0.27	0.39	0.18	0.33
Arousal	0.42	0.39	0.46	0.05	0.18
